# Internal representation of hierarchical sequences involves the default network

**DOI:** 10.1186/1471-2202-11-54

**Published:** 2010-04-27

**Authors:** Baxter P Rogers, Suzanne N Avery, Stephan Heckers

**Affiliations:** 1 Institute of Imaging Science, Vanderbilt University, Nashville, TN, USA; 2 Department of Radiology and Radiological Sciences, Vanderbilt University, Nashville, TN, USA; 3 Department of Biomedical Engineering, Vanderbilt University, Nashville, TN, USA; 4 Department of Psychiatry, Vanderbilt University, Nashville, TN, USA

## Abstract

**Background:**

The default network is a set of brain regions that exhibit a reduction in BOLD response during attention-demanding cognitive tasks, and distinctive patterns of functional connectivity that typically include anti-correlations with a fronto-parietal network involved in attention, working memory, and executive control. The function of the default network regions has been attributed to introspection, self-awareness, and theory of mind judgments, and some of its regions are involved in episodic memory processes.

**Results:**

Using the method of psycho-physiological interactions, we studied the functional connectivity of several regions in a fronto-parietal network involved in a paired image discrimination task involving transitive inference. Some image pairs were derived from an implicit underlying sequence A>B>C>D>E, and some were independent (F>G, H>J, etc). Functional connectivity between the fronto-parietal regions and the default network regions depended on the presence of the underlying sequence relating the images. When subjects viewed learned and novel pairs from the sequence, connectivity between these two networks was higher than when subjects viewed learned and novel pairs from the independent sets.

**Conclusions:**

These results suggest that default network regions were involved in maintaining the internal model that subserved discrimination of image pairs derived from the implicit sequence, and contributed to introspective access of an internal sequence model built during training. The default network may not be a unified entity with a specific function, but rather may interact with other functional networks in task-dependent ways.

## Background

Transitive inference (TI) is the ability to learn associations between items and flexibly use this information. Typical experiments in animals and humans present a series of items in overlapping pairs, e.g. AB, BC, CD, DE, and train via reinforcement that A is to be chosen over B (indicated A>B), B>C, C>D, and D>E. After sufficient training subjects are able to infer the proper order of novel pairs. In particular, B will be correctly chosen over D in the novel pairing B>D. Successful performance of this transitive inference task may require development of an internal representation of the underlying sequence during training, and access to this representation during the post-training test phase.

Previous neuroimaging studies have observed a distributed network of brain regions associated with transitive inference judgments. Multiple studies have reported BOLD response related to transitive inference judgments in the hippocampus, premotor areas, preSMA, the parietal lobe, inferior frontal cortex, and anterior cingulate [1-5]. One study [2] has observed activation of the preSMA, prefrontal cortex, parietal cortex, and temporal cortex for judgments about novel and familiar pairs from the sequence when compared to judgments about similar pairs with no underlying sequence. Additionally, others described bilateral superior and inferior parietal, right DLPFC, bilateral premotor, and SMA activation related to memory maintenance of organized relations similar to those developed during transitive inference experiments [6].

A complete understanding of brain function requires the study of functional connectivity in addition to localization of function, since most cognitive processes involve communication between multiple brain regions. The functional connectivity of the cortical regions associated with TI has been studied in a number of contexts. These regions have been observed to act in concert during the resting state and during a variety of tasks. For instance, a dissociation of cortical networks into attention, memory, and control networks based on resting state functional connectivity has been suggested [7]; the hypothesized fronto-parietal control network in particular overlaps with the regions known to be involved in transitive inference. These networks exhibit temporal and spatial changes during cognitive tasks, such as the auditory oddball [8] and finger tapping at different rates [9]. In summary, the connectivity of the regions involved in transitive inference is likely relevant to task performance, but their underlying connectivity patterns during relational memory tasks are not known.

In this study, we investigated the functional connectivity of parietal, frontal, and prefrontal regions known to be involved in a paired image discrimination task requiring transitive inference. Psycho-physiological interaction models [10] were used to determine how connectivity during image discrimination changed depending on the presence or absence of an underlying sequence, and whether image pairings were learned or novel. We observed that the frontal and parietal regions interacted more strongly with anterior and posterior cingulate default network regions when task performance involved access of an underlying sequence.

## Methods

### Participants and Imaging

**Figure 1:**
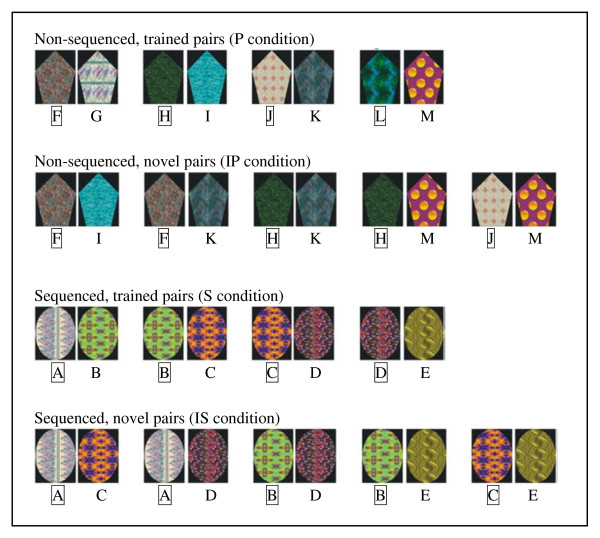
**Cognitive task**. **Four tasks (P, IP, S, IS) were performed in 30-second blocks during fMRI scanning**. Prior to scanning, subjects learned via feedback to discriminate the P and S pairs. During scanning, the subject had to indicate the correct stimulus in each pair via button press (no feedback was given). The pairs presented in the IP and IS conditions were novel and had to be inferred from the previously learned pairs in the P and S condition. For the sequenced set in S and IS, the underlying sequence A>B>C>D>E determined the correct response on all trained and novel pairs. There is no such relationship among pairs for the non-sequenced set in P and IP. Each image has an arbitrary letter label to assist in description; this was not shown to the subjects. For each pair, the correct response is indicated with an outlined letter.

Data from a prior study [2] were used. Sixteen healthy subjects, who gave IRB-approved informed consent as previously described [2], were trained to choose the correct visual pattern from pairs of images presented simultaneously side by side. Two sets of training stimuli were presented (Figure 1). One consisted of four overlapping pairs arranged in sequence: AB, BC, CD, and DE. (The letters represent abstract images that had no inherent relationship prior to training.) In these pairs, A was the correct answer when paired with B; B was correct when paired with C; and so on; in other words the correct answer was determined from the underlying sequence A>B>C>D>E which was not explicitly revealed to the subjects. The control stimuli during training were the unrelated pairs FG, HI, JK, LM. During training all listed pairs were presented and correct choices were indicated with visual feedback. During fMRI scanning, images pairs were presented without feedback to avoid continued learning, and the images were presented in the learned pairings but also in novel pairings of images within each set. Giving correct responses to the novel pairings of images from the sequenced set required transitive inference judgments. The fMRI stimuli were arranged in a 2 × 2 factorial design: (sequence vs. non-sequence) × (learned pairs vs. novel pairs). The four task conditions were S, learned pairs from the sequence; IS, novel pairings from the sequence; P, learned pairs from the non-sequence set; and IP, novel pairings from the non-sequence set. Three-second trials were presented in 30-second blocks in two 5-minutes series. FMRI was performed at 1.5 Tesla using a BOLD-weighted EPI sequence with repetition time of 2500 ms. A more detailed exposition is in the original publication [2].

### Data Analysis

Functional MRI data were re-analyzed in the SPM5 software (Wellcome Department of Imaging Neuroscience, University College London). Functional volumes were co-registered to account for head motion, resampled into the ICBM-152 MNI template space with 2 mm isotropic voxels, and spatially smoothed (8 mm FWHM). A first level voxel-wise univariate general linear model (GLM) was fit for each subject [11], using time as the unit of observation. Functional connectivity was measured using psycho-physiological interactions (PPIs) with a seed region [10]. Deconvolution was not used prior to generating the PPI terms because it has minimal effect for block designs [12].

To generate the regressors for the first level GLM, the seed region time series was first orthogonalized with respect to estimated motion parameters and global signal, to remove the effect of those signals on the connectivity measure. Task regressors were then created for main effects of sequence, inference, and the two-way sequence by inference interaction. These consisted of boxcar indicator variables convolved with a canonical hemodynamic response, and accounted for changes in BOLD signal from block to block. The seed region time series was included as a regressor representing the average level of connectivity between the seed and the voxel in question. Finally, the PPI connectivity regressors representing task-related changes in connectivity consisted of the point-by-point product of the seed region time series with each of the task regressors. The regressors were generated in order: first the task regressors, then the seed, then the two-way interactions sequence by seed and inference by seed, and finally the three-way interaction sequence by inference by seed. The new regressors at each step were orthogonalized with respect to the previous to avoid collinearity in the final design matrix.

Conceptually, this procedure is analogous to estimating connectivity during each task condition separately, then considering connectivity differences between conditions in terms of the main effect and interaction contrasts. The method considers the intrinsic time series variation that underlies the task performance and contributes to trial-to-trial differences [13], as opposed to other sources of variance that have been used to estimate functional connectivity [14-16]. The sequence by seed interaction modeled the increase or decrease of connectivity between seed and voxel in the S and IS conditions relative to the P and IP conditions. The inference by seed interaction modeled the change in connectivity during IS and IP relative to S and P. The sequence by inference by seed interaction modeled a sequence by inference interaction in connectivity values.

For a random effects analysis in the group, second level voxel-wise T-tests of the parameter estimates for sequence by seed, inference by seed, and sequence by inference by seed PPIs were performed. Second level random effects analyses were performed on the estimated psycho-physiological measures at each voxel, including appropriate reaction time covariates to eliminate any behavioral confounds. This was also performed using a general linear model framework, with subject as the unit of observation. The design matrix in each case consisted of a constant vector modeling the mean of the particular PPI, plus a vector of calculated reaction time variables: (RT_S_+RT_IS_)-(RT_P_+RT_IP_) for the sequence by seed PPI; (RT_IS_+RT_IP_)-(RT_S_+RT_P_) for the inference by seed PPI; and (RT_IS_-RT_S_)-(RT_IP_-RT_P_) for the sequence by inference by seed interaction. Estimation used a restricted maximum likelihood technique.

Three seed regions were considered for the connectivity analysis: one in right parietal lobe, one in preSMA, and one in left prefrontal cortex. These were the largest clusters exhibiting a significant transitive inference effect, identified in re-analysis following the methods of the previous work. They also showed responses in all task condition contrasts: sequence vs. non-sequence, inference vs. non-inference, and sequence by inference interaction.

## Results

We measured the functional connectivity between three seed regions in the fronto-parietal control network - right parietal cortex, preSMA, and left prefrontal cortex - and the remainder of the brain to determine whether it varied as a function of cognitive task during discrimination of image pairs. We specifically examined effects of sequence, using images that were or were not members of an underlying 5-item sequence; effects of inference, using image pairs that were or were not learned during pre-scan training; and the sequence by inference interaction. We used the method of psycho-physiological interactions. For each individual, the fMRI signal from a seed region was multiplied point-wise by regressors indicating the different cognitive tasks. This generated new regressors describing the sequence by seed, inference by seed, and sequence by inference by seed psycho-physiological interactions. Inferences on the parameter estimates for these regressors corresponded to tests of whether functional connectivity differed between the tasks.

**Figure 2:**
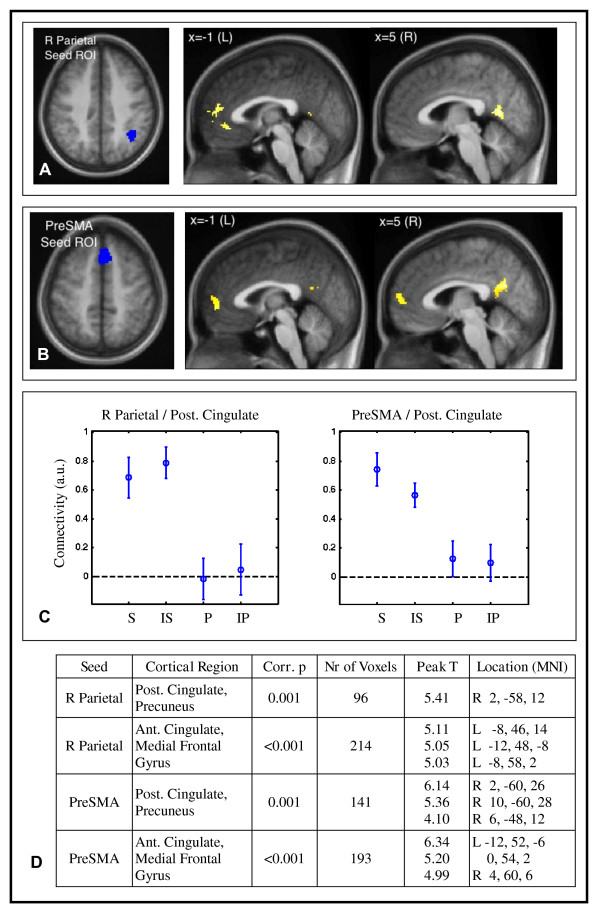
**Fronto-parietal connectivity and sequence representation**. **The fronto-parietal network interacted more strongly with the default network during tasks that used an internal sequence representation**. During scanning, participants responded to learned and novel pairs from a sequenced set and a non-sequenced set (Figure 1). Connectivity depended on the presence or absence of the underlying sequence. ***(A) Midline areas showed increased connectivity with the right parietal seed during sequence tasks***. Functional connectivity was higher during S and IS conditions than P and IP, a positive Sequence by Parietal Seed psycho-physiological interaction. The orange/yellow colored voxels exhibited this interaction in a second-level analysis (p < 0.01). ***(B) Midline areas also showed increased connectivity with the preSMA seed during sequence tasks***. Colored voxels exhibited a positive Sequence by PreSMA Seed psycho-physiological interaction (p < 0.01). ***(C) Connectivity between the fronto-parietal network and the posterior cingulate was high during sequence tasks S and IS, low during non-sequence tasks P and IP***. Right: connectivity between right parietal seed and posterior cingulate from (A). Left: connectivity between PreSMA seed and posterior cingulate from (B). Error bars indicate the standard error of the mean. ***(D) The midline areas are within the default mode network***. The table gives cluster coordinates in the MNI atlas space, corresponding to maps in (A) and (C). Voxels individually were p < 0.001 uncorrected, and reported clusters were significant at p < 0.01 corrected for multiple voxel comparisons based on cluster extent (one-tailed tests). The clusters were located in anterior cingulate, medial frontal gyrus, posterior cingulate, and precuneus, areas associated with the default network.

The fronto-parietal network interacted more strongly with anterior and posterior midline regions during tasks that accessed an internal sequence representation (Figure 2). This was observed for both the right parietal and the preSMA seed regions, which showed stronger connectivity with midline areas including anterior and posterior cingulate, medial prefrontal cortex, and precuneus during the S and IS conditions compared to the P and IP conditions. Figure 2 shows these regions of the brain that exhibited a significant sequence by seed interaction for the right parietal seed (Figure 2A) and the preSMA seed (Figure 2B). Figure 2C shows specific examples: the connectivity between the right parietal seed and the posterior cingulate region indicated in Figure 2A was calculated for each cognitive task from the parameter estimates of the psycho-physiological interaction model. Connectivity between these two regions was high during the sequence tasks S and IS, but near zero for the non-sequence tasks P and IP. This was also true for connectivity between the preSMA seed and the posterior cingulate region shown in Figure 2B. Figure 2D lists all the regions that showed the sequence by seed interaction, with their locations. Only voxels significant at p < 0.001 (one-tailed) were considered. Among these, we retained only those in contiguous clusters large enough to be significant at p < 0.01 (one-tailed) corrected for multiple comparisons based on the assumptions of random field theory [17,18].

These anterior and poster midline regions coincide with the default network, a set of brain regions that typically exhibit reduced fMRI signal during complex or attention-demanding tasks compared to simple rest or fixation [19,20]. The distance between the centers of the clusters reported in Figure 2 and the centers of the default network regions reported in a recent test-retest analysis of resting state data [21] ranged from 4-19 mm, suggesting a large degree of spatial coincidence. The default network includes anterior and posterior cingulate; medial frontal cortex; precuneus; lateral temporo-parietal areas; and portions of the medial temporal lobe. In fact we did observe sequence by seed psycho-physiological interactions in some of these additional regions at a statistical threshold of p < 0.01 (voxelwise uncorrected).

In the resting state, negative correlations are typically present between the default network and the fronto-parietal network that contains our three seed regions when data are preprocessed using our techniques [22-24]. In our case, correlations between the two networks were generally positive during the sequence conditions, for example Figure 2C.

In a post-hoc analysis, we observed that explicit awareness of the underlying sequence may affect the relationship between the fronto-parietal network and the default network. Eleven of the 16 subjects demonstrated post-experiment explicit awareness of the underlying sequence A>B>C>D>E as determined by post-test interview. The sequence by seed interaction in connectivity for the right parietal seed (Figure 2D) was stronger in the sequence-aware subjects (p < 0.01 in 8 voxels in precuneus and 3 in medial frontal gyrus, considering only those voxels with an overall sequence by seed interaction at p < 0.005).

**Figure 3:**
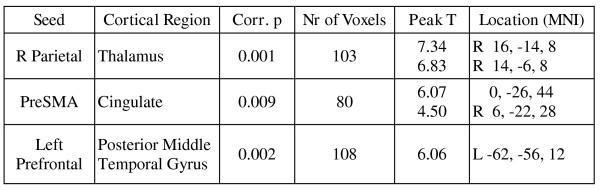
**Fronto-parietal connectivity and inference**. **Some cortical regions showed stronger connectivity with the fronto-parietal seed ROIs during responses to learned pairs, versus to novel pairs**. The table shows clusters of voxels with a significant Inference by Seed psycho-physiological interaction (p < 0.01 corrected), all of which showed higher connectivity with the corresponding seed ROI during the S and P conditions compared to IS and IP.

Some brain regions showed stronger connectivity with the fronto-parietal seed regions during responses to learned pairs, versus responses to novel pairs (Figure 3). These were the right thalamus, the mid cingulate gyrus, and the left posterior middle temporal gyrus, all of which showed stronger connectivity with one of the seed regions during the S and P tasks compared to IS and IP (p < 0.01 corrected at the cluster level). The table in Figure 3 lists these regions and their locations.

Our regions of interest showed a significant transitive inference effect in their responses to the stimuli [2], but we saw no sequence by inference by seed interaction that would suggest a transitive inference effect in connectivity. Because of this, we further analyzed the relationship between the behavioral data and functional connectivity with the right parietal seed region. The transitive inference effect was apparent in subject response times, which were generally slower for the novel pair sequence condition IS relative to the learned pair sequence condition S, beyond the amount that would be expected strictly due to novelty as represented by the difference between the novel pair and learned pair non-sequence conditions IP and P. This can be quantified in terms of the reaction times for each condition as the transitive inference reaction time RT_TI _= (RT_IS _- RT_S_) - (RT_IP _- RT_P_). The analogous metric of functional connectivity is the sequence by inference by seed psycho-physiological interaction, which was estimated as described previously and can be considered conceptually as the transitive inference connectivity effect C_TI _= (C_IS _- C_S_) - (C_IP _- C_P_).

**Figure 4:**
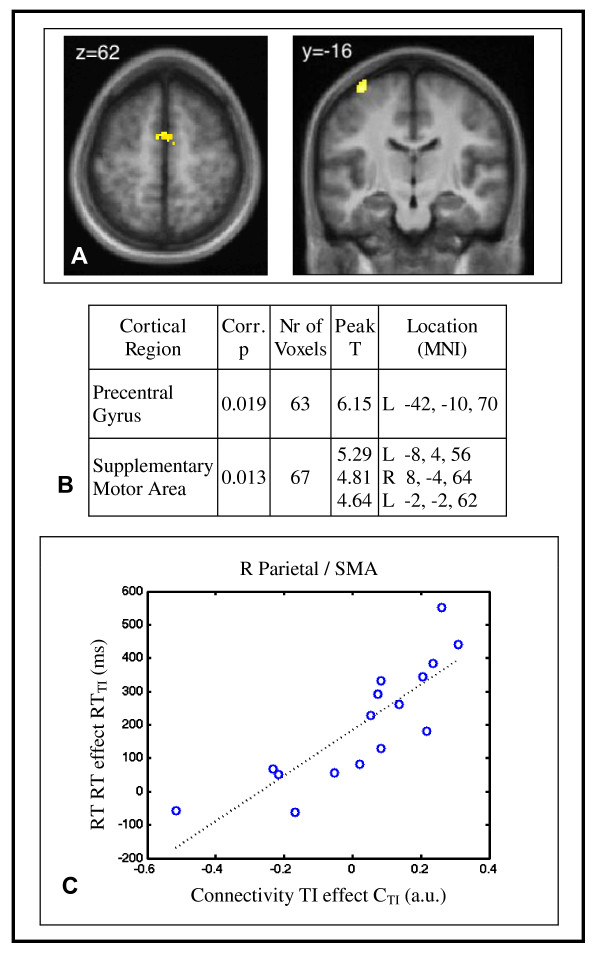
**Connectivity/behavior correlations**. **Individual differences in connectivity between the right parietal seed region and the motor network partially explained the behavioral transitive inference effect**. The behavioral TI effect was calculated from the reaction times for the four conditions: (RT_IS_-RT_S_)-(RT_IP_-RT_P_), which is the additional response time for the novel sequence (transitive inference) pairs relative to the learned sequence pairs, above and beyond the portion attributable to novelty only as determined from the non-sequence pairs. The connectivity TI effect was the parameter estimate for the sequence by inference by seed psycho-physiological interaction, analogous to (C_IS_-C_S_)-(C_IP_-C_P_) with C the connectivity between each voxel and the seed. ***(A) The behavioral and right parietal connectivity transitive inference effects were correlated in the bilateral supplementary motor area and left precentral gyrus***. Significant positive correlation between the sequence by inference by seed psycho-physiological interaction and the behavioral TI effect was present in the colored voxels in a second-level analysis (p < 0.05). ***(B) The areas of correlation were the bilateral supplementary motor area and left precentral gyrus***. The table gives cluster coordinates in the MNI atlas space corresponding to the map in (A). Voxels were p < 0.001 uncorrected, and reported clusters were significant at p < 0.05 corrected for multiple comparisons based on cluster extent (one-tailed tests). The clusters were located in areas associated with motor planning and execution. ***(C) The relationship between connectivity and behavioral TI effects was approximately linear***. The plot shows the values from the SMA.

Individual differences in connectivity with the right parietal seed region partly explained the behavioral transitive inference effect (Figure 4). We observed this in bilateral supplementary motor area and left precentral gyrus, regions involved in the planning and execution of voluntary movement. Figure 4A shows the statistical parametric map of the areas with a significant correlation between the behavioral and right parietal connectivity transitive inference effects. Figure 4B lists the local maxima in the significant clusters, with their locations; voxels significant at p < 0.001 (one-tailed) were considered, and only those in contiguous clusters large enough to be significant at p < 0.05 (one-tailed) corrected for multiple comparisons were retained. Figure 4C shows the TI reaction time plotted against the TI connectivity effect between the right parietal seed and the SMA cluster of Figure 4A.

## Discussion

Two lines of research have implicated the precuneus and posterior cingulate regions where we observed sequence-related changes in connectivity: episodic memory retrieval, and resting-state imaging of the default mode network. In studies of memory retrieval, these areas have shown higher BOLD response to remembered stimuli than to correctly rejected novel stimuli; they have also shown higher response to specifically recollected or deeply encoded stimuli compared to merely familiar or shallowly encoded stimuli: [25] for a review and meta-analyses, [26] for a specific example of precuneus involvement in episodic memory. On the other hand, these same areas are part of the default mode network which has been defined as those brain regions which show reduced BOLD response during complex or goal-directed cognitive tasks compared to a resting baseline condition [20] and anti-correlations with fronto-parietal networks at rest [22] and during cognitive tasks [27]. We observed that connectivity between the fronto-parietal network and the default network was increased during the two task conditions S and IS that could benefit from sequence knowledge. Choosing the correct image for sequenced pairs requires reference to an internal representation of the sequence, which was never explicitly given during training; the imaging results suggest that the default network contributed to transitive inference via introspective access of an internal sequence model built on the past training experiences. That increased connectivity between the two networks was apparent in the S condition (memorized premise pairs) as well as the IS condition (novel pairs requiring inference) suggests that the sequence representation was accessed even for familiar pairs, when they contained images from the sequence. Overall, our results indicate that retrieval of the sequence representation involved these midline regions specifically; rather than showing the anti-correlations associated with the resting state, the midline default network contributed directly to the retrieval task because it was needed for successful sequence retrieval.

People perform differently on this type of transitive inference task depending on their explicit awareness of the underlying sequence. Performance by unaware subjects was generally worse in a number of studies that grouped subjects by awareness post-hoc [28-30], although a direct correlation between awareness and performance has not always been apparent [31,32]. Most likely multiple strategies are in play [33]. In our sample, awareness was fairly high as indicated by 82% overall accuracy on BD trials, compared to 45%-65% for unaware subjects in other studies. Therefore it is possible that the high connectivity measured between task active regions and default mode regions in sequence trials reflects conscious access of a sequence representation. If so, this connectivity should be diminished or absent in subjects who remain unaware of the sequence but still perform above chance, for example by associative weighting during learning [34]. The data set presented here was not large enough to test this hypothesis effectively; however, results of the small post hoc analysis are intriguing. The larger difference in connectivity between sequence and non-sequence conditions in aware subjects hints that sequence awareness affects the relationship between the fronto-parietal system and the default network.

More aspects of the relationship between fronto-parietal and default networks during transitive inference may be discovered in patient groups. For example, transitive inference is affected in schizophrenia[35,36], a disorder which is thought to involve disrupted connectivity[37]. Schizophrenic patients perform as well as controls on all image pairs but the novel BD transitive inference pair [4]. If the connected relationship between fronto-parietal and default networks indeed contributes to access and maintenance of a sequence representation, this might be expected to diminish in the patients who fail to draw on a flexible representation of the sequence. Affected connectivity between fronto-parietal and default mode cortical networks has in fact been observed in schizophrenia. Controls show less correlation between the networks than patients in an independent component analysis when correlations are generally positive [38], and more negative values of connectivity than patients when global signal removal has caused the correlation values to be negative [39]. This pattern suggests a weaker differentiation of the two networks in patients, which could correspond with a lack of distinctive resources for patients to draw on for flexible use of the sequence.

Our study has some methodological limitations which we do not consider critical to the results. We measured functional connectivity based on fMRI time series data acquired while different conditions were presented in 30-second blocks. The signals that underlie functional connectivity measurements, specifically those signals that have identified the fronto-parietal and default networks in other studies, occur at frequencies below 0.1 Hz. A 30-second block contains 3 or fewer cycles of such signals. For this reason the ability to measure functional connectivity from 30-second segments of data is probably reduced. However, 45-second blocks have proven sufficient in other studies, e.g. [40,41]. In fact, the sequence by seed and sequence by inference by seed psycho-physiological interactions in this study were determined from contiguous 60-second segments of the time series because of the order of conditions (S, IS, P, IP, S, IS, P, IP). A second consideration is the measurement of functional connectivity from data acquired during continuous performance of a task. Past work indicates that the low-frequency signals are measurable in this situation [13], are behaviorally meaningful [42], and may exhibit different patterns of connectivity than the resting state [9,27,40]. Thirdly, we removed the estimated global signal prior to calculating connectivity; this preprocessing step is known to affect connectivity values [24,43,44]. Because of this, we do not overintepret the absolute values of connectivity shown in Figure 2. In particular, we observed zero connectivity between some regions during the non-sequence conditions, but that does not necessarily imply the absence of a functional relationship. The primary finding was the connectivity *difference *between sequence and non-sequence conditions, which were affected equally by pre-processing.

## Conclusions

Our observation of increased connectivity between fronto-parietal and midline default network regions suggest that the midline regions were recruited to support memory retrieval. This is similar to prior episodic retrieval experiments, and different from findings of decreased BOLD response during experiments studying language, visual search, and spatial attention.

## Authors' contributions

BPR and SH conceived of the study. BPR directed the connectivity analysis and drafted the manuscript. SNA assisted with background research and data analysis, and evaluated the subject awareness measures. SH participated in study design and evaluation of results, and helped to draft the manuscript. All authors critically read and approved the final manuscript.
